# Cytosolic CARP Promotes Angiotensin II- or Pressure Overload-Induced Cardiomyocyte Hypertrophy through Calcineurin Accumulation

**DOI:** 10.1371/journal.pone.0104040

**Published:** 2014-08-04

**Authors:** Ci Chen, Liang Shen, Shiping Cao, Xixian Li, Wanling Xuan, Jingwen Zhang, Xiaobo Huang, Jianping Bin, Dingli Xu, Guofeng Li, Masafumi Kitakaze, Yulin Liao

**Affiliations:** 1 State Key Laboratory of Organ Failure Research, Department of Cardiology, Nanfang Hospital, Southern Medical University, Guangzhou, China; 2 Department of Pharmacy, Nanfang Hospital, Southern Medical University, Guangzhou, China; 3 Cardiovascular Division of the Department of Medicine, National Cerebral and Cardiovascular Center, Osaka, Japan; Osaka University Graduate School of Medicine, Japan

## Abstract

The gene ankyrin repeat domain 1 (Ankrd1) is an enigmatic gene and may exert pleiotropic function dependent on its expression level, subcellular localization and even types of pathological stress, but it remains unclear how these factors influence the fate of cardiomyocytes. Here we attempted to investigate the role of CARP on cardiomyocyte hypertrophy. In neonatal rat ventricular cardiomyocytes (NRVCs), angiotensin II (Ang II) increased the expression of both calpain 1 and CARP, and also induced cytosolic translocation of CARP, which was abrogated by a calpain inhibitor. In the presence of Ang-II in NRVCs, infection with a recombinant adenovirus containing rat Ankrd1 cDNA (Ad-Ankrd1) enhanced myocyte hypertrophy, the upregulation of atrial natriuretic peptide and β-myosin heavy chain genes and calcineurin proteins as well as nuclear translocation of nuclear factor of activated T cells. Cyclosporin A attenuated Ad-Ankrd1-enhanced cardiomyocyte hypertrophy. Intra-myocardial injection of Ad-Ankrd1 in mice with transverse aortic constriction (TAC) markedly increased the cytosolic CARP level, the heart weight/body weight ratio, while short hairpin RNA targeting Ankrd1 inhibited TAC-induced hypertrophy. The expression of calcineurin was also significantly increased in Ad-Ankrd1-infected TAC mice. Olmesartan (an Ang II receptor antagonist) prevented the upregulation of CARP in both Ang II-stimulated NRVCs and hearts with pressure overload. These findings indicate that overexpression of Ankrd1 exacerbates pathological cardiac remodeling through the enhancement of cytosolic translocation of CARP and upregulation of calcineurin.

## Introduction

Cardiac ankyrin repeat protein (CARP) binds to various proteins, such as titin/connectin, myopalladin [1], and calsequestrin [2], and it plays a critical role in the maintenance of sarcomere integrity, stretch sensing, and excitation-contraction coupling under physiological and pathological conditions. Similar to the genes for natriuretic peptides and β-myosin heavy chain (β-MHC), the gene for CARP (termed ankyrin repeat domain 1: Ankrd1) was also identified as a fetal gene, expression of which is augmented in both animals and humans with cardiac hypertrophy [3], [4] and heart failure (HF) [5], [6]. Natriuretic peptides have been shown to have cardioprotective effect [7]. In contrast, the role of Ankrd1/CARP in cell growth remains controversial. Although there is clinical evidence that Ankrd1 mutations are involved in the pathogenesis of hypertrophic and dilated cardiomyopathy [8]–[10], there is no consensus about whether CARP enhances or attenuates cardiac hypertrophy.

Subcellular distribution of CARP is thought to be associated with its different function [11], while the cytosolic protease calpain 3 has been reported to be responsible for cytosolic retention of CARP [12]. It appears that Ankrd1/CARP can function as pro-hypertrophic gene in the myofibril by gain of function and as an anti-hypertrophic gene in the nucleus by suppression of cardiac genes expression [11], [13]. A recent study revealed that hypertrophic cardiomyopathy (HCM) associated mutations in Ankrd1 (P52A, T123M, and I280V) have an increased binding ability of CARP to titin and myopalladin [9], while overexpression of either wildtype or HCM related mutant Ankrd1 (P52A or I280V) in cardiomyocytes reduced myocyte contractility [14]. In addition, it has been proposed that calpains influence signaling pathways that are involved in myocardial hypertrophy, including those for calcineurin [15], [16]. Therefore, we postulated that an increased cytosolic translocation of CARP could be a mediator of calpain-related signal transduction processes. Moreover, CARP is involved in calcium-dependent signaling [2], while increased binding of CARP to titin and myopalladin has been suggested to induce myocardial hypertrophy [9]. These findings suggest the hypothesis that CARP might accelerate the progression of cardiac hypertrophy.

Although substantial evidence indicates that evaluation of Ankrd1/CARP expression may be helpful for diagnostic or prognostic assessment of cardiac hypertrophy, it is unknown whether Ankrd1/CARP could also be a therapeutic target. Considering that overproduction of angiotensin II (Ang II) is responsible for cardiac hypertrophy and upregulation of Ankrd1/CARP, it seems reasonable for Ankrd1/CARP to be a potential target of Ang II receptor 1 blockers (ARB). Accordingly, we used Ang II-stimulated cultured cardiomyocytes and mice with transverse aortic constriction (TAC) to examine the following issues: (1) the influence of Ankrd1/CARP on cardiac hypertrophy; (2) whether Ankrd1/CARP modulates calcineurin and nuclear factor of activated T cells (NFAT) and (3) whether the ARB olmesartan medoxomil prevents the upregulation of Ankrd1/CARP in response to Ang II or pressure overload.

## Materials and Methods

All procedures were performed in accordance with our Institutional Guidelines for Animal Research and the investigation conformed to the Guide for the Care and Use of Laboratory Animals published by the US National Institutes of Health (NIH Publication No. 85-23, revised in 1996). The study was approved by the ethics review board of Southern Medical University. The concentrations or dose of olmesartan and its active form RNH-6270 were decided according to previous reports [17] and our preliminary experiments.

### Cell culture

The neonatal rats were sacrificed by 2% isoflurane inhalation and cervical dislocation. Isolation and culture of ventricular cardiomyocytes was performed as described previously [18]. The confirmation of cell type was performed using immunochemistry assay (antimyoactin antibody, Wuhan Boster Biological Technology., LTD, China). Cells were cultured for 4 days and then treated with 0.1–1 µM Ang II or 1 µM calpain inhibitor 1 (Sigma Aldrich), or RNH-6270 1 µM (RNH, active metabolite of olmesartan, provided by Daiichi Sankyo, Tokyo), or recombinant adenovirus containing the rat Ankrd1 cDNA (Ad-Ankrd1) or adeno-associate virus (AAV) carrying short hairpin RNA targeting Ankrd1 (sh-Ankrd1). Cell surface area was also measured by staining with rhodamine phalloidin and diamidino-2-phenylindole dihydrochloride (DAPI). Briefly, the cells were fixed with 4.0% paraformaldehyde in PBS, permeabilized in 0.1% Triton X-100 in PBS, and stained with rhodamine phalloidin (5 µg/ml, Invitrogen) and DAPI (5 mg/mL, Beyotime) by standard immunocytochemical techniques. At least 30 random cells from each group were measured by the Image J software.

### Construction of recombinant adenovirus carrying Ankrd1 or AAV containing silenced Ankrd1

The full-length cDNA of rat Ankrd1 was inserted into the vector pUC57, and then subcloned into the shuttle vector pDC316-mCMV-EGFP. The Ankrd1 cDNA clones were sequenced completely to confirm the absence of cloning artifacts and mutation. The Ad-MAX system was used for the generation of recombinant adenovirus carrying Ankrd1 cDNA or empty vector (pEGFP). Briefly, pDC316-mCMV-EGFP-Ankrd1 and virus backbone plasmid pBHGloxdelIE13cre were co-transfected into cultured HEK293 cells by using lipofectamine 2000 (Invitrogen), then the recombinant adenovirus were collected and amplified in HEK293. The overexpression of Ankrd1 was achieved by transfecting cultured neonatal rat ventricular cardiomyocytes (NRVCs) with the recombinant adenovirus (multiplicity of infection (MOI) = 10) or by myocardial injection of 5×10^11^ adenovirus particles containing Ankrd1 or control vector into three points of the left ventricular wall.

AAV-mediated gene delivery was also employed. pAAV2/9-CMV-ZsGreen vectors carrying short hairpin RNA targeting Ankrd1 (sh-Ankrd1) or scramble were created by a professional company (Biowit, Shenzheng, China). For in vitro transfection, pAAV2/9-CMV-ZsGreen-shAnkrd1 or scramble virus particles (5×10^5^ viral genomes/cell) were added in cultured NRVCs. After transfection for ninety-six hours, infection efficiency and silencing effect were evaluated using a fluorescence microscopy and western blot, respectively. For in vivo transduction, pAAV2/9-CMV-ZsGreen-sh-Ankrd1 or scramble virus particles (1×10^11^ viral genomes/ml) were administered by direct injection in the left ventricular free wall (2 sites, 10 µl/site) in mice at 4 weeks old using a syringe with a 30-gauge needle, and four weeks later, sham or TAC surgery was performed.

Transduction efficiency of in vivo gene transfer by adenovirus or AAV was assessed by EGFP fluorescence (510 nm) in cryosectioned heart slices using a fluorescence microscopy.

### Pressure overload model

C57BL/6 male mice (8–10 weeks old, weighing 22–25 g, provided by the Animal Center of Southern Medical University) were anesthetized with a mixture of xylazine (5 mg/kg, intraperitoneally) and ketamine (100 mg/kg, intraperitoneally), and the adequacy of anesthesia was monitored from the disappearance of pedal withdrawal reflex. Pressure overload model was created by transverse aortic constriction (TAC) as described elsewhere [18], [19]. Some of the TAC mice were randomized to treatment with olmesartan (Daiichi Sankyo, Tokyo, 10 mg/kg/d added in the food) or vehicle alone for 4 weeks. The mice were sacrificed by overdose anesthesia (pentobarbital sodium 150 mg/kg intraperitoneally) and cervical dislocation.

### Polymerase chain reaction analysis (PCR) and Western blot

Total RNA of homogenized murine whole heart or cultured cardiomyocytes was isolated using Total RNA Kit II (Omega) according to the protocol provided by the manufacturer. Conventional or quantitative real-time PCR using a Quantitect SYBR Green RT-PCR kit (QIAGEN) and an Applied Biosystems 7500 system targeting the genes of Ankrd1, ANP, β-MHC, calpain 1, early growth response 1 (egr-1), β-actin and GAPDH, was performed. Primers were showed in Table 1 and 2.

**Table 1:** Sequences of primers for regular PCR.

Transcripts	Forward primer (5′–3′)	Reverse primer (5′–3′)	Product size (bp)
ANP (rat)	GGCTCCTTCTCCATCACCAA	TGTTATCTTCGGTACCG	458
ANKRED1 (rat)	CGGGATCCATGATGGTTTTTCGAGTAGAGG	GGCCTCGAGTCAGAACGTAGCTATGCGC	960
Egr-1 (rat)	AGCCTTCGCTCACTCCACTA	GACTCAACAGGGCAAGCATAC	184
GAPDH (rat)	ACCAACTGCTTAGCCCCCC	GCATGTCAGATCCACAACGG	281

**Table 2:** Sequences of primers for real-time PCR.

Transcripts	Forward primer (5′–3′)	Reverse primer (5′–3′)	Product size (bp)
β-MHC (rat)	GCTACCCAACCCTAAGGATGC	TCTGCCTAAGGTGCTGTTTCA	196
ANP (rat)	CTCCGATAGATCTGCCCTCTTGAA	GGTACCGGAAGCTGTTGCAGCCTA	216
β-actin (rat)	ATCGTGCGGGACATCAAGG	CAGGAAGGAGGGCTGGAACA	180
ANP (mouse)	CGGTGTCCAACACAGATC	TCTTCTACCGGCATCTTTC	71
β-actin (mouse)	TGGACAGTGAGGCAAGGATAG	TACTGCCCTGGCTCCTAGCA	121
Calpain 1 (rat)	TGCCATGTTCCGTGCTTTCA	GACCGTAAAAACGTGGCTGC	178
GAPDH (rat)	GATGCCCCCATGTTTGTGAT	GGTCATGAGCCCTTCCACAAT	216

Proteins were extracted from the cultured cardiomyocytes or their mitochondria, or the murine heart. Immunoblotting was performed by using antibodies against CARP (Santa Cruz), calcineurin (Abcam). Blotting of β-actin or GAPDH (Santa Cruz) was used as a loading control.

### Immunofluorescence assay

Immunofluorescence staining was used to examine the cellular distribution of the CARP proteins and NFAT. Location of CARP and NFAT (Abcam) in the cardiomyocytes was detected with a confocal microscopy.

### Statistical analysis

Data are expressed as the mean ± SEM. Statistical significance was analyzed using Student’s unpaired *t*-test or one-way ANOVA, followed by Bonferroni’s correction for post hoc multiple comparisons. In all analyses, *P* values<0.05 were considered to indicate statistical significance.

## Results

### Ang II upregulates Ankrd1/CARP in cultured cardiomyocytes

**Figure 1:**
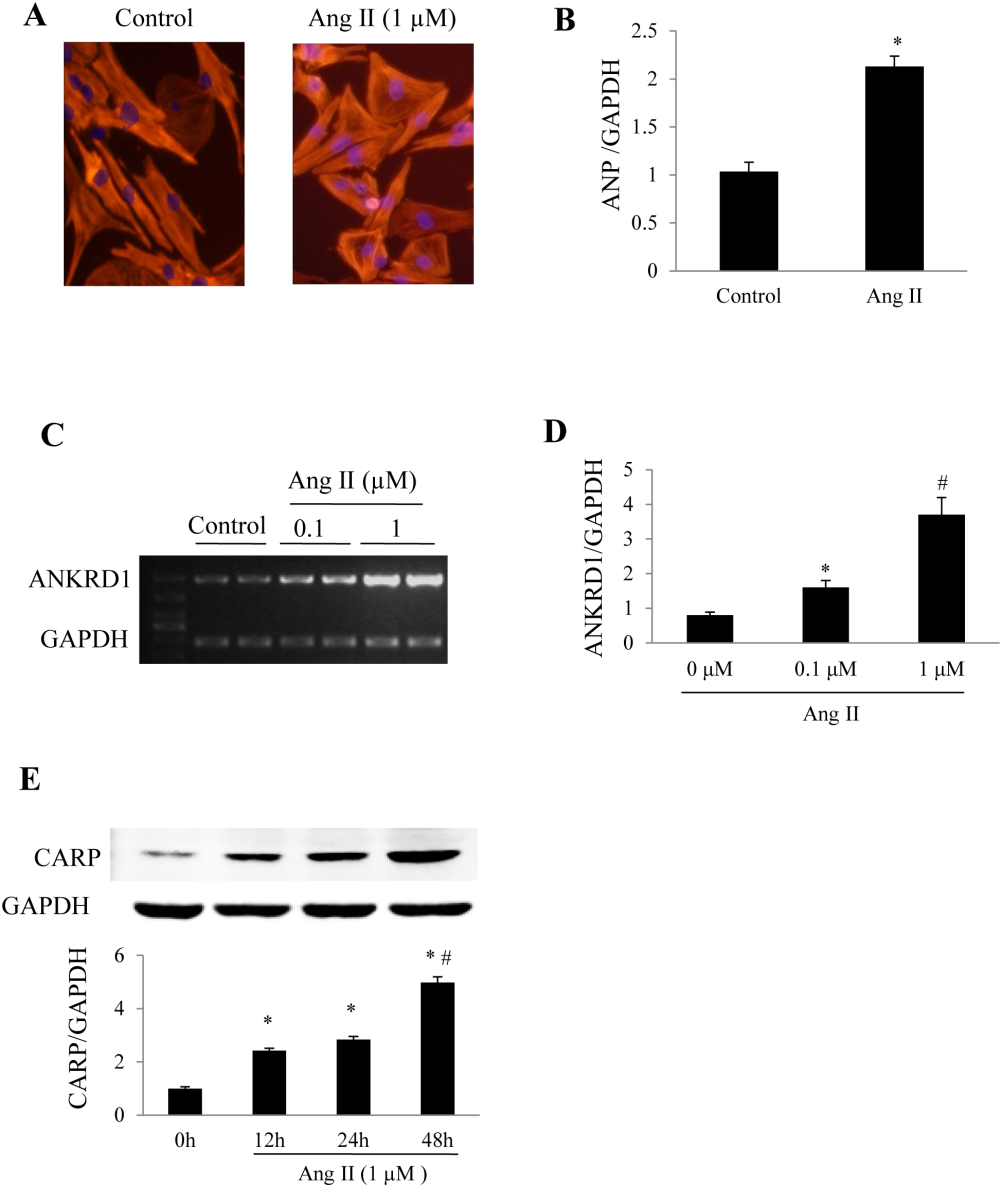
Ankrd1/CARP upregulation in response to Angiotensin II stimulation. ***(A)*** Representative images of cultured neonatal rat ventricular cardiomyocytes (NRVCs) stained with rhodamine phalloidin and 4′, 6-diamidino-2-phenylindole dihydrochloride (DAPI), and the cell size increased when treated with Angiotensin II (Ang II 1 µM) for 24h. ***(B)*** Hypertrophic marker atrial natriuretic peptide (ANP) detected by Real-time PCR using GAPDH as an internal control (*P<0.05 vs. the control group). Expression changes of Ankrd1 in response to Ang II in cultured NRVCs were detected by PCR ***(C)*** or real-time PCR ***(D)***. ***(E)*** Western blot analysis of time-dependent changes of CARP expression in NRVCs exposed to Ang II. *P<0.05 vs. Ang II-untreated group, #*P*<0.01 vs. Ang II 24 h group. Repeat times n = 3. Data are mean ± SEM.

In cultured NRVCs, stimulation with Ang II for 24 h increased the cell surface area and upregulated ANP (Fig. 1 A and B). Ang II stimulation markedly increased the expression of Ankrd1 mRNA detected by routine PCR (Fig. 1C) and quantitative real-time PCR (Fig. 1D), as well as causing a time-dependent increase of CARP protein (Fig. 1E). These findings indicate that the myocardial expression pattern of Ankrd1/CARP is similar to that of natriuretic peptides during pathological stimulation.

### Cytosolic translocation of CARP in response to Ang II or infection of Ad-Ankrd1 in cultured NRVCs

**Figure 2:**
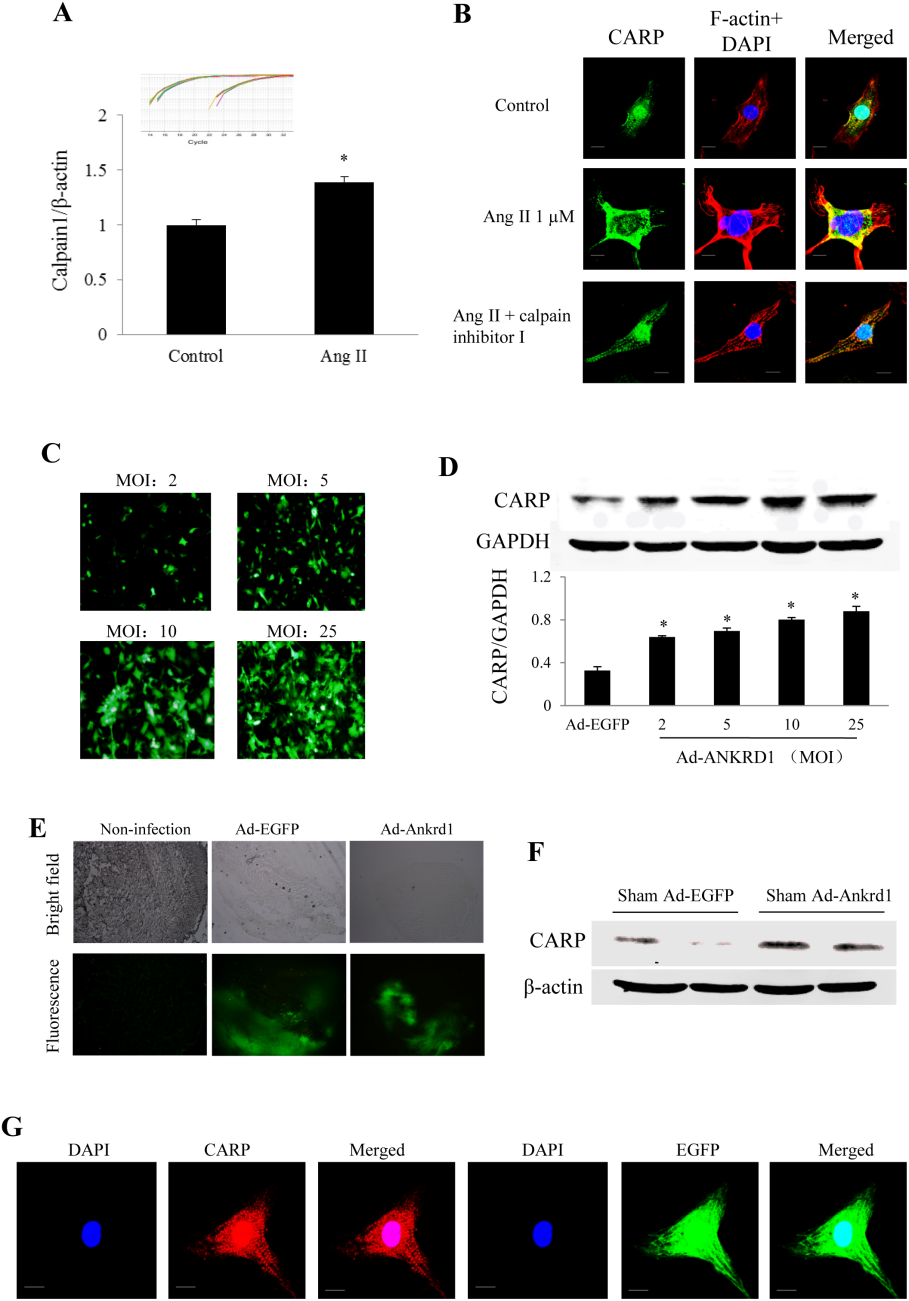
Subcellular translocation of CARP in response to treatment with Ang II or Ad-Ankrd1. ***(A)*** Expression changes of calpain 1 in response to Ang II stimulation in cultured neonatal rat ventricular cardiomyocytes (NRVCs) detected by real-time PCR, the insert represents amplification curve of calpain 1 and β-actin, *P<0.05. ***(B)*** Confocal microscopic subcellular distribution of CARP (green) in response to Ang II stimulation in NRVCs. Immunostaining with F-actin (red) was used to confirm the cardiomyocytes, and DAPI (blue) was used to stain the nuclei. Bar = 10 µm. ***(C)*** Dose-dependent infective efficiency of Ad-Ankrd1 in cultured NRVCs detected by the green fluorescence of co-expressed EGFP. MOI: multiplicity of infection. ***(D)*** Western blot analysis of CARP protein levels in response to different dose of Ad-Ankrd1 infection (*P<0.01 vs. Ad-EGFP group, n = 3). ***(E)*** Representative EGFP fluorescence microscopic photos of LV myocardium at 1 week after intramyocardial injection of vehicle (non-transfection), Ad-EGFP or Ad-Ankrd1 delivery. ***(F)*** Western blot analysis of CARP protein levels in response to intramyocardial injection of Ad-EGFP or Ad-Ankrd1 at 1 week after sham operation. ***(G)*** Subcellular location of forced Ankrd1 expression in Ad-Ankrd1 transfected cardiomyocytes. The green fluorescence was emitted from the report gene EGFP which was constructed in adenovirus carrying Ankrd1. Bar = 10 µm.

Ang II stimulation markedly increased the expression of calpain 1 mRNA (Fig. 2A). CARP was mainly localized to the nucleus under basal conditions, cytoplasmic translocation of CARP was noted when Ang II treatment for 24 h in NRVCs, which was abolished by a calpain 1 inhibitor (Fig. 2B), suggesting that cytosolic retention of CARP contributes to Ang II-induced cardiomyocyte hypertrophy. Then we infected NRVCs with Ad-Ankrd1. After incubation for 48 h, the transfection efficiency of Ad-Ankrd1 or Ad-EGFP and the subcellular localization of Ankrd1 in cultured cardiomyocytes were evaluated by confocal fluorescence microscopy. It was found that the number of Ad-Ankrd1-transfected cells (Fig. 2B) and the level of CARP protein (Fig. 2C) both increased in a MOI-dependent fashion. We noted that the forced overexpression of Ankrd1 was detected in both the cytoplasm and the nucleus (Fig. 2G).

### Forced Ankrd1 overexpression aggravates cardiomyocyte hypertrophy in response to Ang II stimulation

**Figure 3:**
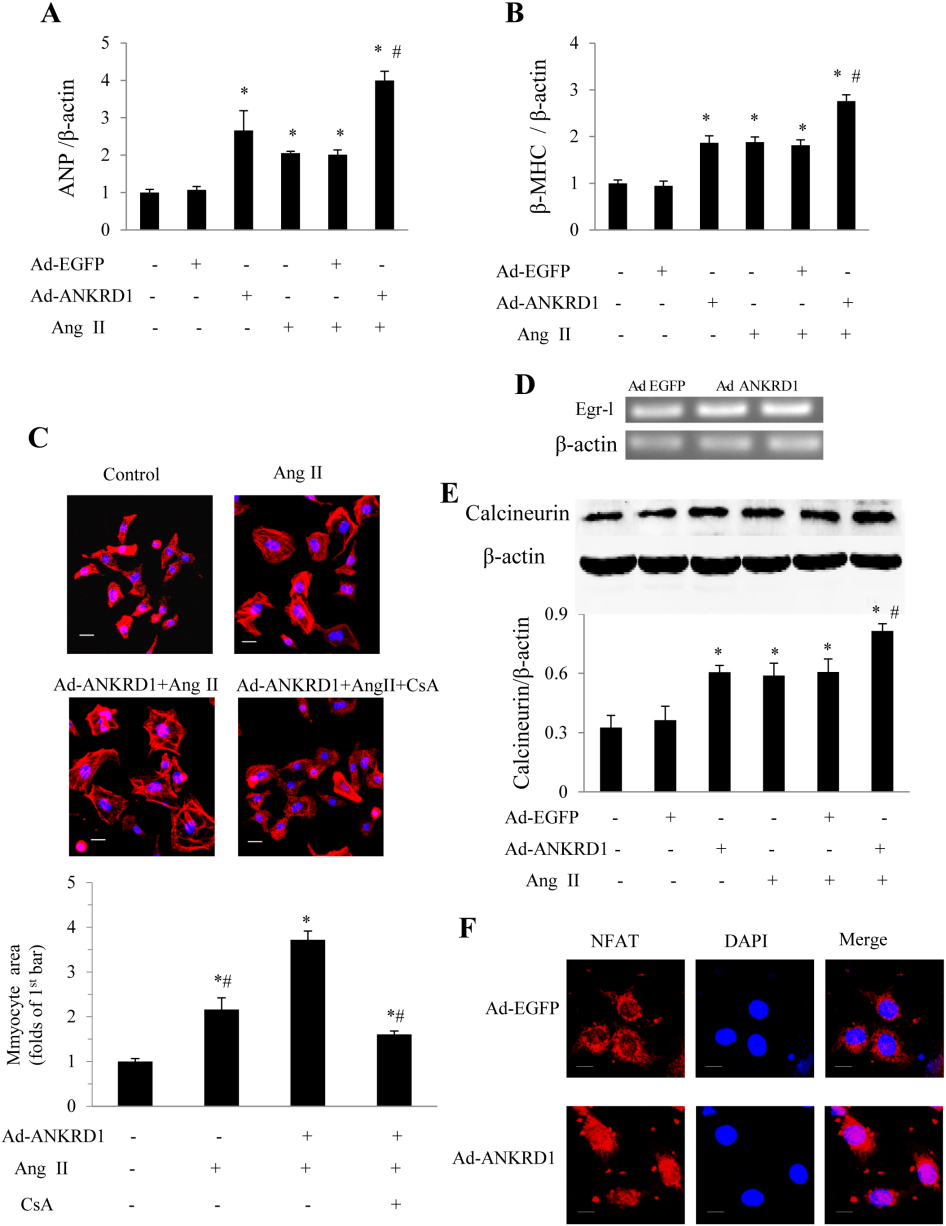
Effect of Ad-Ankrd1 transfection on myocyte hypertrophy in NRVCs. Hypertrophic markers of ANP ***(A)*** and β-MHC ***(B)*** gene expressions in response to Ang II stimulation in the presence of Ad-Ankrd1 or Ad-EGFP (10 MOI) were detected by Real-time PCR (*P<0.01 vs. control, #*P*<0.01 vs. Ad-EGFP + Ang II, n = 5). ***(C)*** Representative pictures of cultured cardiomyocytes used for calculation of cross section area and quantitative results (*P<0.01 vs. control (1^st^ bar), #*P*<0.01 vs. Ad-Ankrd1+ Ang II, n = 100 in each group). ***(D)*** PCR results of early growth response 1 (egr-1) in response to Ad-Ankrd1 treatment. ***(E)*** Western blot analysis of calcineurin in cardiomyocytes in response to different treatment (*P<0.01 vs. control (1^st^ bar), #*P*<0.01 vs. Ad-EGFP + Ang II group). ***(F)*** Nuclear translocation of nuclear factor of activated T cells (NFAT) induced by Ad-Ankrd1 transfection in the absence of Ang II. Every experiment was repeated at least 3 times. Dose of Ang II was 1 µM. Scale bar = 10 µm for panel *C* and *E*. Data are mean ± SEM.

The hypertrophy-associated fetal genes ANP and β-MHC were significantly upregulated in Ad-Ankrd1 infected cardiomyocytes both in the absence and the presence of Ang II stimulation (Fig. 3A and B). In addition, overexpression of Ankrd1 significantly enhanced the Ang II-stimulated increase of cell surface area (Fig. 3C). CARP was reported to upregulate egr-1 [20] which participates calcineurin-NFAT signal pathway [21]. It has been well documented that the calcineurin/NFAT pathway plays a pivotal role in myocardial hypertrophy [22]. Therefore, we examined erg-1 mRNA, calcineurin and NFAT protein levels. We noted that Ankrd1 overexpression significantly upregulated erg-1 (Fig. 3 D). Western blot analysis showed that overexpression of Ankrd1 led to a significant increase of calcineurin (Fig. 3E) and to an increased expression and nuclear translocation of NFAT (Fig. 3F) even in the absence of Ang-II stimulation. The increase of cell surface area stimulated by Ad-Ankrd1 and Ang II was blocked by the calcineurin inhibitor cyclosporine A (CsA, Sigma) (Fig. 3C). These results indicate that forced Ankrd1 overexpression promotes cardiomyocyte hypertrophy via the calcineurin-NFAT pathway.

### Effects of Ankrd1 overexpression or silence on cardiac hypertrophy

**Figure 4:**
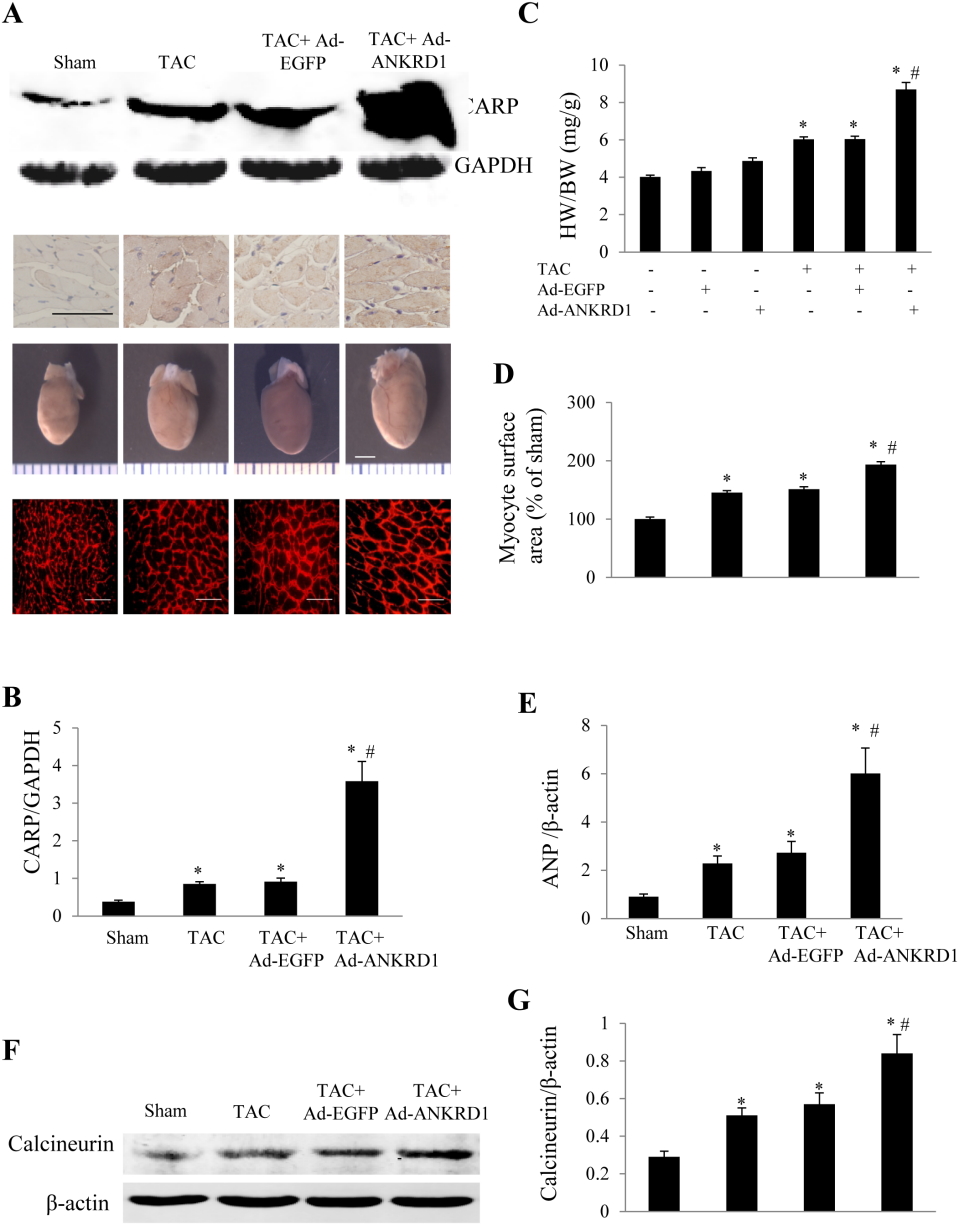
Myocardial injection of Ad-Ankrd1 in mice promotes cardiac hypertrophy. Cardiac overexpression of Ankrd1 was achieved by myocardial injection of Ad-Ankrd1 at 2 weeks after TAC, and the mice in each group were sacrificed at 4 weeks after the initial surgery. ***(A)*** Representative pictures of Western blot for CARP (upper panel), immunohistochemistry of myocardial CARP (2^nd^ line, scale bar = 50 µm), whole hearts (3^rd^ line, scale bar = 3 mm) and myocyte cross-sectional area stained with rhodamine-conjugated wheat germ agglutinin (low panel, scale bar = 30 µm) from each group. ***(B)*** Expression levels of CARP in each group. ***(C)*** HW/BW ratio at 4 weeks after surgery in different groups. ***(D)*** Myocyte surface area was calculated from 100 cells in each group. ***(E)*** ANP gene expression detected by Real-time PCR using β-actin as an internal control. ***(F)*** Western blot analysis of calcineurin expression in heart from each group. ***(G)*** Semi-quantitative analysis of calcineurin expression. For panel *B-E, G*, *P<0.01 vs. sham, #*P*<0.01 vs. TAC + Ad-EGFP, n = 4–9 in each group. Data are mean ± SEM.

In the mice receiving sham operation, about 60% infection efficiency was obtained for the whole heart at 1 week after intramyocardial injection of Ad-Ankrd1 manifested by green fluorescence in cardiomyocytes under fluorescence microscopy as well as a significantly higher CARP expression level than in Ad-EGFP group (Fig. 2E and F). Cardiac overexpression of Ankrd1/CARP in TAC mice was achieved by intramyocardial injection of Ad-Ankrd1 at 2 weeks after TAC. Another 2 weeks later, total myocardial CARP expression was dramatically higher in Ad-Ankrd1-infected TAC mice than in Ad-EGFP-infected TAC mice (Fig. 4A and B), and overexpressed CARP largely localized in the cytoplasm (Fig. 4A). Furthermore, myocardial overexpression of Ankrd1 was found to promote TAC-induced cardiac hypertrophy (Fig. 4A). The heart weight/body weight (HW/BW) ratio was significantly higher in Ad-Ankrd1-TAC mice than in Ad-EGFP-TAC mice (8.69±0.38 mg/g in vs. 6.04±0.15 mg/g, *P*<0.01, Fig. 4C). Similarly, the TAC-induced increase of cardiomyocyte surface area and ANP expression was markedly enhanced by overexpression of Ankrd1 (Fig. 4A, D and E, all *P*<0.01). Myocardial calcineurin expression was higher in TAC mice than in shame group, while it was further enhanced in TAC mice treated with Ad-Ankrd1 (Fig. 4F and G). These findings indicated that myocardial overexpression of Ankrd1 exacerbates cardiac hypertrophy.

**Figure 5:**
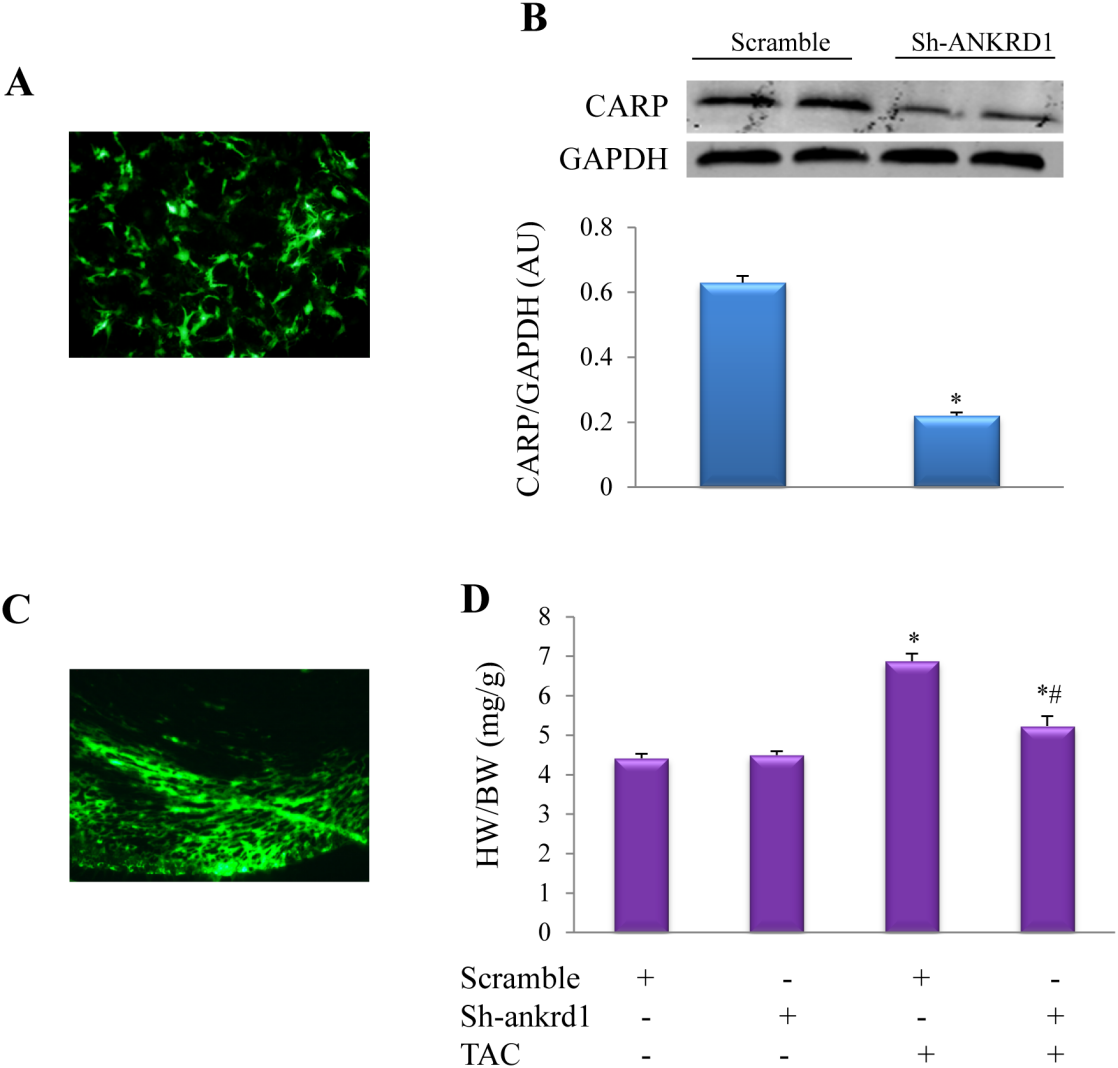
Myocardial injection of AAV-sh-Ankrd1 in mice inhibits cardiac hypertrophy. ***(A)*** Infective efficiency of AAV-sh-Ankrd1 in cultured NRVCs for 96 h detected by the green fluorescence of co-expressed EGFP (zsGreen). ***(B)*** Western blot analysis of CARP protein levels in response to AAV-sh-Ankrd1 or AAV-scramble infection (*P<0.01 vs. scramble group, n = 5). ***(C)*** Representative fluorescence microscopic pictures of left ventricular myocardium 5 weeks after intramyocardial AAV-sh-Ankrd1 delivery. ***(D)*** Heart weigh/body weight ratio (HW/BW) in TAC or Sham mice treated with AAV-scramble or AAV-sh-Ankrd1. *P<0.01 vs. the corresponding Sham (TAC -) group, #*P*<0.01 vs. TAC + scramble group, n = 5 in each group. TAC or Sham was persisted for 1 week.

Silencing effect of sh-Ankrd1 was confirmed in cultured NRCs. AAV2/6 transfection for 96 h in cardiomyocytes led to about 60% transfection efficiency, while western blot showed that CARP was downregulated by 3 folds (Fig. 5A and B). AAV2/6 transfection for 5 weeks in murine heart resulted in more than 50% transduction efficiency (Fig. 5C). One week after TAC, we noted that myocardial transduction of AAV-sh-Ankrd1 could inhibit cardiac hypertrophy evidenced by that the HW/BW ratio was significantly smaller in AAV-sh-Ankrd1-TAC mice than in the scramble-TAC mice (Fig. 5D).

### Olmesartan attenuates cardiac hypertrophy in TAC mice through down-regulation of Ankrd1/CARP

**Figure 6:**
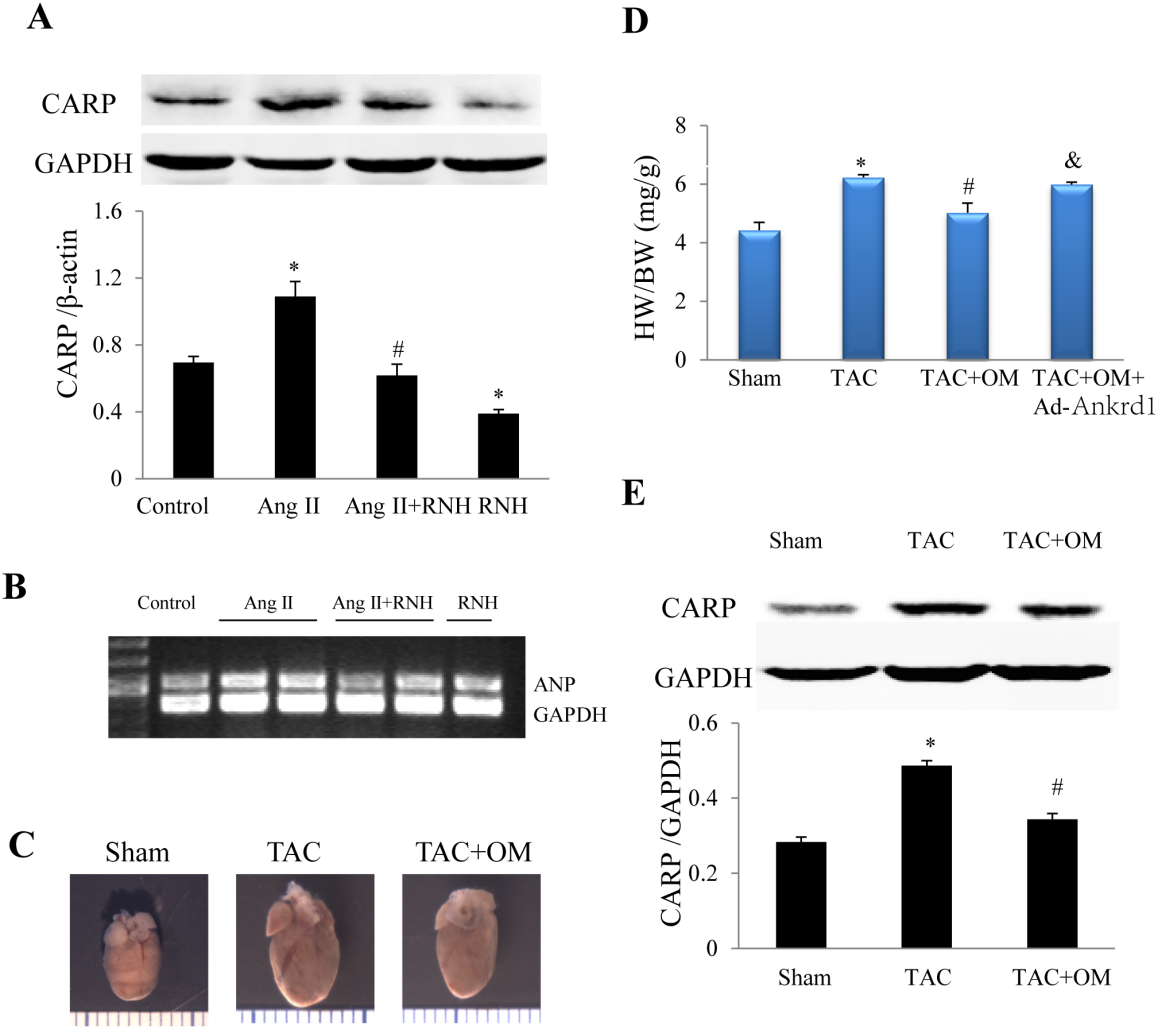
Olmesartan downregulates CARP and ameliorates cardiomyocyte hypertrophy. ***(A)*** Treatment with RNH6270 (RNH 1 µM, active form of olmesartan) reduced Ang II (1 µM) -induced increased of CARP protein in NRVCs (*P<0.05 vs. control, #*P*<0.05 vs. Ang II. n = 3). ***(B)*** ANP mRNA expression in NRVCs exposed to Ang II (1 µM) stimulation in the presence/absence of RNH (1 µM). ***(C)*** Representative picture of whole heart from different group (scale bar = 2 mm). ***(D)*** HW/BW ratio was significantly lower in TAC mice treated with olmesartan medoxomil (OM) in comparison with untreated TAC mice, while addition of Ad-Ankrd1 partially blocked the antihypertrophic effect of OM. (*P<0.01 vs. Sham, #*P*<0.01 vs. TAC, &*P*<0.05 vs. TAC + OM, n = 4–6 in each group). ***(E)*** Olmesartan treatment for 4 weeks reduced the myocardial expression of CARP in TAC mice (*P<0.05 vs. Sham, #*P*<0.05 vs. TAC). Data are mean ± SEM.

Considering the above findings that upregulation of myocardial Ankrd1/CARP in response to Ang II stimulation and pressure overload had a detrimental influence on myocyte hypertrophy, we investigated whether Ankrd1/CARP was a target of olmesartan, which is a selective angiotensin II type 1 receptor antagonist. Treatment with 1 µM RNH-6270 significantly prevented the Ang II-stimulated upregulation of CARP protein and ANP gene expression in cultured cardiomyocytes (Fig. 6A and B). Four weeks later, olmesartan significantly reduced the TAC-induced cardiac hypertrophy (Fig. 6C), and the HW/BW ratio in olmesartan-treated TAC mice was significantly smaller than in vehicle-treated TAC group, while addition of Ad-Ankrd1 partially blocked the antihypertrophic effect of olmesartan (*P*<0.05 or 0.01, Fig. 6D). CARP expression was significantly lower in TAC mice treated with olmesartan than in untreated TAC mice (Fig. 6E).

## Discussion

Although it has been demonstrated that Ankrd1/CARP expression is upregulated in response to myocardial damage and dysfunction regardless of the etiology [3], [4], [6], and elevation of Ankrd1/CARP expression is interpreted as an indicator of an unfavorable clinical outcome, it remains unclear whether myocardial overexpression of Ankrd1 in cardiac hypertrophy is causally related to the development of a malignant cardiac phenotype or whether it is merely an adaptive response that delays the progression of cardiac hypertrophy. This study provided both in vitro and in vivo evidence that myocardial overexpression of Ankrd1/CARP promotes cardiac hypertrophy. Cytosolic translocation of CARP induced by either pathological stress or forced overexpression, also contributed to the malignant cardiac phenotype. Another interesting finding was that Ankrd1/CARP is a potential pharmacological target for the treatment of cardiac remodeling. Our present findings support the concept that Ankrd1/CARP is a versatile factor which exerts pleiotropic effects on cardiomyocytes at multiple levels.

Arimura et al. detected three HCM-associated missense mutations (P52A, T123M, and I280V) in Ankrd1, all of which showing increased binding of CARP to titin and myopalladin in the I band region of the sarcomere [9], while a recent study by Crocini et al. showed that overexpression of either wildtype or mutant Ankrd1 (P52A and I280V) in cardiomyocytes led to a reduced cell contractility [14]. These lines of evidence imply that sarcomeric translocation of CARP and increased binding to titin and myopalladin are necessary for hypertrophy enhancement by CARP under pathological stress. The subcellular localization of CARP under physiological and pathophysiological conditions remains obscure or even controversial. Some studies indicated that CARP is a cardiac-restricted nuclear protein with a pivotal role in fetal development [23] or in failing and non-failing hearts of human [5], [24], but CARP was also detected in the cytoplasm rather than the nucleus of cardiomyocytes from HF patients [6], [25] or even in neonatal rat cardiomyocytes [9]. Generally, it is believed that CARP is both cytosolic and nuclear localization [8], [10] which functions as stretching-sense component in the cytoplasm and a co-factor of cardiac gene expression in the nuclei, and their subcellular localization depends on physiological and pathological stresses. The present study showed that CARP was mainly located in the nucleus of neonatal cardiomyocytes under physiological conditions and underwent translocation to the cytoplasm in response to stimulation by Ang II. Moreover, we found that forced overexpression of CARP increased its cytosolic distribution and had several detrimental effects on cardiomyocytes. In adult mice receiving sham or TAC treatment, myocardial CARP is preferentially localized in cytoplasm. Therefore, it seems that cytoplasmic translocation of CARP in response to a pathological insult contributes to myocardial hypertrophy. Although there is no consensus about the subcellular distribution of CARP in pathological states [8], [9], [11], [24], our results are in agreement with the findings of a previous clinical study that CARP immunoreactivity was localized to the cytoplasm in diseased myocardium and was barely detectable in the nucleus [6].

To date, the in vivo evidence concerning the role of CARP in myocardial hypertrophy are scant. HCM associated Ankrd1 mutations result in an increased binding of CARP to sacomeric titin and myopalladin as well as a dual intracellular localization within sarcomere and nucleus [9]. On the other hand, overexpression of Ankrd1 has been reported to inhibit the phenylephrine-stimulated enlargement of neonatal rat cardiomyocytes [10], [26], which is contradictory to our present findings and a consensus that “gain” of function (such as overexpression) for a sarcomeric gene usually results in hypertrophy [13]. One explanation for such discrepancy is the different intracellular localization of CARP dependent on the experimental conditions. Duboscq-Bidot et al reported that more nuclear translocation of CARP occurred upon phenylephrine stimulation [10], which is in contrast to our finding that cytosolic translocation occurred upon Ang-II stimulation. It seems that nuclear Y-box-binding protein (YB-1) can stimulate cell proliferation [27], whereas nuclear CARP can bind with YB-1 and inhibit its activity [11], implying that nuclear localization of CARP exerts anti-hypertrophic role. As the first in vivo evidence on the role of Ankrd1 in myocardial hypertrophy, Song et al recently reported that cardiac-specific overexpression of Ankrd1 attenuated pressure overload- or isoproterenol-induced cardiac hypertrophy in mice [26], which is in contrast to our present findings. The reasons for this discrepancy are unclear. Although in vivo evidence about the influence of CARP on the growth of cardiomyocytes is scant, there are several lines of evidence which suggest that Ankrd1/CARP promotes non-cardiomyocyte cell growth rather than inhibiting it. For example, CARP was reported to promote neurite outgrowth in F11 cells [28], as well as the growth of cancer cells [29] and neovascularization in tissue wounds [30].

As reviewed by Mikhailov et al. [11], since the identification of Ankrd1/CARP in 1995, most investigations have focused on the induction of Ankrd1 expression in cultured primary cardiomyocytes or non-cardiomyocyte cell lines. Thus, there is little in vivo evidence about the effects of Ankrd1/CARP-mediated pathways in cardiomyocytes. Forced overexpression of wild type Ankrd1 in cultured NRVCs was reported to inhibit ANP gene promoter activity [23], while there are reports showed that no any significant influence on ANP levels was found when Ankrd1 or even HCM-associated Ankrd1 mutants were overexpressed [14]. These findings are not in agreement with the in vivo evidence that significant up-regulation of Ankrd1/CARP is associated with re-activation rather than inhibition of ANP in various types of HF [31]. In addition, we noted that forced overexpression of Ankrd1/CARP markedly upregulated calcineurin, which further challenges the idea that Ankrd1/CARP acts as a negative regulator of cardiac gene expression [24].

In this study, we found that both Ang II stimulation and pressure overload promoted the cytosolic accumulation of CARP, which in turn activated the calcineurin-NFAT pathway, suggesting that CARP may be a useful target for the treatment of myocardial hypertrophy. It deserves to investigate how CARP activates calcineurin-NFAT pathway. Coincidently, Boengler et al. reported that CARP upregulates egr-1 [20], while egr-1 has been reported to participate calcineurin-NFAT signal pathway-induced myocardial hypertrophy [21], which in agreement with our findings in this study. We also found that olmesartan, a selective AT1 receptor blocker (ARB), attenuated cardiac hypertrophy in TAC mice at least partially through down-regulation of CARP expression and consequently alleviating the accumulation of calcineurin. In agreement with our findings, it has been reported that losartan, another ARB, markedly inhibits the increase of calcineurin activity in cardiomyocytes stimulated by Ang II [32]. In mice, total Ankrd1 knockout or cardiac-restricted overexpression did not produce an abnormal phenotype [26], [33], which is in agreement with our finding that in vivo overexpression of Ankrd1 alone did not induce cardiac hypertrophy when no pathological stress was added, suggesting that Ankrd1 is redundant and plays pathological stress-dependent role. These lines of evidence suggest that pharmacological inhibition of Ankrd1/CARP would not cause severe side effects. Therefore, it would seem reasonable to attempt the development of pharmaceutical CARP antagonists for the treatment of cardiac hypertrophy.
